# A phenomenological study of nurses experience about their palliative approach and their use of mobile palliative care teams in medical and surgical care units in France

**DOI:** 10.1186/s12904-020-0536-0

**Published:** 2020-03-20

**Authors:** Agnès Oude Engberink, Maryse Mailly, Valerie Marco, Daniele Bourrie, Jean-Pierre Benezech, Josyane Chevallier, Sandrine Vanderhoeven, Remy Crosnier, Gérard Bourrel, Béatrice Lognos

**Affiliations:** 1 University of montpellier CEPS platform, site Saint Charles, 34000 Montpellier, France; 2 Maison de Santé Pluriprofessionnelle Universitaire Avicenne, 2 rue IBN Sinai dit Avicenne, 66330 Cabestany, France; 3 Department of Palliative Care, CHU Montpellier, Montpellier, France; 4 Mobile Palliative Care Team, CHU Montpellier, Montpellier, France; 5 Department of General Medecine, University of Montpellier, avenue du Doyen Gaston Giraud, 34000 Montpellier, France

**Keywords:** Patient-centered palliative approach, Mobile palliative care team, Phenomenology, Physician nurse balance, Recognition at work

## Abstract

**Background:**

Despite a broad consensus and recommendations, numerous international reports or studies have shown the difficulties of implementing palliative care within healthcare services. The objective of this study was to understand the palliative approach of registered nurses in hospital medical and surgical care units and their use of mobile palliative care teams.

**Methods:**

Qualitative study using individual in depth semi-structured interviews and focus group of registered nurses. Data were analyzed using a semiopragmatic phenomenological analysis.

Expert nurses of mobile palliative care team carried out this study. 20 registered nurses from three different hospitals in France agreed to participate.

**Results:**

Nurses recognize their role as being witnesses to the patient’s experience through their constant presence. This is in line with their professional values and gives them an “alert role” that can anticipate a patient-centered palliative approach. The physician’s positioning on palliative care plays a key role in its implementation. The lack of recognition of the individual role of the nurse leads to a questioning of her/his professional values, causing inappropriate behavior and distress.

According to nurses, “rethinking care within a team environment” allows for the anticipation of a patient-centered palliative approach. Mobile Palliative Care Team highlights the major role of physicians-nurses “balance” while providing personal and professional support.

**Conclusions:**

The Physician’s positioning and attitude toward palliative approach sets the tone for its early implementation and determines the behavior of different staff members within healthcare service. “Recognition at work”, specifically “recognition of the individual role of nurse” is an essential concept for understanding what causes the delay in the implementation of a palliative approach. Interprofessional training (physicians and nurses) could optimize sharing expertise. Registered nurses consider MPCT as a “facilitating intermediary” within the healthcare service improving communication. Restoring a balance in sharing care and decision between physicians and other caregivers lead care teams to an anticipated and patient-centered palliative approach according to guidelines.

## Background

The evaluation conducted in France by la Direction Générale de l’Offre de Soins (Directorate of Health Care Supply) of the plan from 2008 to 2012 and the report concerning Palliative Care [1] underscored the primary mission of the Mobile Palliative Care Teams (MPCT): the implementation of a Palliative Approach (PA) by care-givers within hospital care units. In France, a MPCT is a multidisciplinary team (nurses, physicians, psychologist …) within a hospital which offers a consultation service to other care units. Its members do not provide directly patients care but advise and support teams.

The report of the Commission on End-of-Life in France found “an inability of specialists to engage in a PA within their units. It showed that 80% of physicians had never been specifically trained” [2]. The American Society of Clinical Oncology recommending the early merging of standard care and palliative care, found a gap between recommendations and practices [3]. An American study which evaluated current hospital-based palliative care programs using the recommendations from the Center to Advance Palliative Care in seven hospitals [4], identified several barriers: limitations in palliative care skills, the understanding of the patient’s social and spiritual concerns and overall communication among a team of care providers, which emphasize the need to expand educational and training programs. A Canadian Society of Cancer report noted that the use of palliative care services by medical caregivers remained low [5]. In 2012, one third of oncologists sent their terminal cancer patients to a palliative care service in Canada; while in within the region of Paris, only 3 oncologists out of 150 were trained in palliative care [2].

However, a number of studies showed that the early application of a PA provide beneficial results for patients with cancer: better quality of life, less depression, less hospitalisations and higher rate of survival [6, 7]. If the interest of palliative care has not yet been discussed, why is there reluctance in implementing it early on? An Italian study with 283 nurses working in medical, surgical and intensive-care units, showed that the nurses who were given the necessary room to provide the best care they could deliver exhibited less cases of burn-out and development of strategies to improve nurse satisfaction in working were recommended [8]. Another qualitative study on the causes of nurses stress identified high workloads, unavailability of physicians, unsupportive management and interpersonal issues [9].

In 2016, the MPCT from the Montpellier’s CHU took on 608 palliative care patients (2266 interventions within 58 medical and surgery care-units for adults) and delivered 1053 h of basic and follow-up training for the caregivers [10]. Despite positive feedback from caregiver teams, MPCT nurses reported difficulties by nurses in the care-units in grasping palliative care and replicating what they had learned from their training. One Swiss phenomenological study about the utility of MPCT showed difficulties in initiating a partnership, the nurses preferring to delegate complex care to the nurse of the MPCT [11]. A Belgian study defined caregiver resistance in implementing a PA as “the unconscious refusal to put attitudes or learned actions into practice when faced with a patient in need of palliative care” and identified six factors: feelings of powerlessness, the lack of training, the need to protect oneself from stress, personal beliefs and the quality of social and organizational support [12]. A French study showed that in spite of a 5-year training program, the implementation of a PA was tardy; 49% of nurses feared that the use of a PA would cause anxiety in the patient and 90% found it difficult to choose the right moment [13]. A cross-sectional Survey of 363 nurses in a multispecialty hospital showed that overall level of nurses’ knowledge about palliative care was poor [14]. For Gamblin et al. [15] the resistance of the caregivers came from a discrepancy between a normative ideal and the actual reality.

In Norway, nursing homes and home-care managers requested the mobile teaching team’s service for supervision for caregivers who felt anxious. Expert nurses helped be more secure and confident when faced with situations of bereavement and were able to guide nurses of care-units althrough the complexities of practice within «workplace learning » [16].

The diverse data poses numerous questions: Why do nurses encounter reluctance to the implementation of palliative care despite its effectiveness? Are these difficulties psychological, organizational, and/or managerial? How can MPCTs help them?

## Methods

### Aim

The objective of the work initiated by Montpellier’s MPCT was to understand the PA of the nurses in the medical and surgical care-units of 3 hospitals in the South of France and the circumstances and impact of the use of MPCTs, using a phenomenological approach focused on the lived experience.

### Design

A qualitative phenomenological study was chosen to describe and understand the experiences of nurses within complex PC situations. This report were guided by COREQ [17].

### Recruitment and characteristics of participants

A letter was distributed by the health services to Registered Nurses (RN) facing palliative care, informing them of the research and of the upcoming study. The nurses were recruited from the medical and surgical care units in three different locations in the south of France, on a voluntary basis through authorization from the head of healthcare: CHU of Montpellier, CHU of Nîmes, and the hospital of Sète. Patients with both curative and palliative needs were cared for on these wards. We used purposive sampling to obtain a diversity of nurses’ experiences across various characteristics such as age, sex, the type of health service, years of practice and specific training. We applied the principle of data saturation without pre-defining the number of interviews.

### Data collection

The first collection method comprised of phenomenological interviewing that took place in person, to understand the experience in depth. The second took the form of focus groups, to look at the interaction between participants and their common experiences. An interview guide (Table 1) including open-ended phenomenological questioning focused on lived experience was developed. Two qualitative research methodologists verified the appropriateness, as well as the intelligibility of the questions, after the two initial test sessions were conducted with nurses. Follow-up prompts were designed to lead participants to recount their personal experience. The same guide was used for the focus group. The three interviewers (MPCT nurses) received preliminary training on phenomenological reformulation in order to carry out in-depth interviews. An experienced moderator (qualitative methodologist) facilitated the focus groups. Participant characteristics were noted and the conditions surrounding the interviews was taken into account for the analysis: the time, place, position of those involved and context. The interviewer introduced himself as a researcher working in the field of palliative care. The interviews were recorded with a TASCAM Dr-100MKII digital recorder. The sound quality was tested and deemed sufficient quality for recording. The interviewer made sure to create an atmosphere of confidence so that responses would be spontaneous. The recordings were transcribed verbatim by two secretaries. Each received a number in order to remain anonymous. We did not plan to collect non-verbal data.

**Table 1:** Interview Guide

**In a few words, what does palliative care mean to you?**	
**I In the healthcare service where you encounter palliative care: How would you say it goes in general? What happens?**	
Follow-up: *What would you say is the place of primary care nurses within the context of PC? How do you imagine it?* *Are you satisfied? Do you like your role?* *What does a palliative approach mean to you?*	
**II Do you remember the last patient that you had who went through palliative care with you: Describe the situation. Think about the day, the place and who you were with. What happened? What were your thoughts at the moment? What did you do?**	
Follow-up: *Do you remember what you experienced in the situation? What thoughts came into your mind? What did you do and why?* *Which problems did you need to solve? What did you have at your disposal from your personal experience that helped you? (see if she makes reference to experience, professional values, knowledge, techniques, biographical lived events, other colleagues? Which other colleagues?)* *Did you feel that you had a particular need in that moment or that you were lacking anything? If so, did it cause you to suffer in any way?* *What did you take from this experience?*	
**III With regards to this personal experience that you have just spoken about: The litterature says that despite a solid knowledge of palliative care by healthcare workers, patients do not always benefit from a palliative approach. What do you think about that?** **How would you judge the partnership with MPCT? With the nurses in the MPCT?**	
Follow-up: *In what ways specifically do you see this collaboration? Do you think this collaboration is possible?* *Can you tell me if you have found difficulties in this partnership? What are they?* *Have you had an experience where a palliative approach was not implemented? Do you remember your thoughts or emotions at that moment?* *Do you recall a situation where a palliative approach was put into place? (wait for the answer before asking the second part of the question) Did you experience a particular feeling? What was it? Did you feel enriched? In what way?*	
**IV In light of everything that you’ve said so far, what could shared care mean*for you*?**	
Follow-up: *Would you be able to integrate this idea into your daily practice?*	
**Do you have anything to add?**	

### Data analysis

Semiopragmatic Phenomenology is a descriptive method for categorizing lived experience recorded in interview transcripts. The first steps of this analysis (Table 2) were performed according to a constant comparison process [18] to build the categories, completed by a semiopragmatical data interpretation procedure inspired by C.S. Peirce [19]. In this method, the analyst takes into account all the semiotic elements of a text, including linguistic and contextual clues. First, empirical categories emerge by constant comparison. Secondly semiopragmatical analysis allowed the logical ordering of these empirical categories according to Peirce’s theory of signs [20, 21]. Typically, as a result of this ordering, the conceptually densest category (i.e., of the highest level in the hierarchy of signs) commanded the meaning of the phenomenon at play. The same analysis method was used for the different methods of data collection.

**Table 2:** Semiopragmatic Analysis Steps according to Peirce’s theories

Accurate transcription of the recordings (French: *verbatims*).	
Identifying the most relevant elements of the preexisting context.	
General intuitive reading, followed by targeted reading.	
Dividing text into meaning chunks and then into the first themes.	
Identifying all indexical, textual and contextual signifiers (indices) for a first categorization.	
Categories increase in level of generalization through constant comparison	
Arranging those categories according to their logical inter-relationships.	
Constructing the meaning of an emerging phenomenon via a general proposition. Meaning organization though logical ordering according to the hierarchy of signs	

### Ethics approval and consent to participate

Participants signed an informed consent form. The respondents were informed that their responses would remain confidential and anonymous as they their name would not be aggregated with their personal identification details. They were also informed that they could stop participating in the study at any point.

We asked the privacy and compliance officer of the university hospital of Montpellier for a IRB review. Since no data were collected regarding the participants health statuses, there was no obligation to seek approval from an ethical committee in France. However, the university hospital of Montpellier recorded the material in accordance with all french ethical regulations (ref: MR-003).

## Results

### Participants

Among the 20 nurses that agreed to participate, 18 actually participated in the study. The semi-structured interviews were conducted in a quiet office during the work hours of the participants. This explains why two were not able to be interviewed as they were not available. 11 individual interviews were conducted between February 2016 and march 2017 on-site in three different hospitals. As the interview test did not reveal any problems, the test data was included in the final dataset. The interviews lasted between 35 and 90 min. Data saturation was reached at the 9th individual interview; the two supplemental interviews did not add any new information. The focus group with 7 RNs from different medical and surgical services took place in January 2017 and lasted 1 h and 45 min in the MPC unit. The atmosphere surrounding the interviews was friendly. The characteristics of the participants are reported in Table 3. The transcribed text of the individual interviews was numbered from I0 to I10, and the transcribed text from the participants in the focus groups, from F1 to F7.

**Table 3:** Participant Characteristics

	Age	Marital and Family Status	Year and diploma obtained	Professional Experience	Training
**E0**	30	Domestic partner No children	2009	Medical service I at CHU of city A for 5 years	3 days of institutional training in the palliative approach
**E1**	27	Domestic partner No children	2012	Medical service I at CHU of city A for 2 years	3 days of institutional training in the palliative approach
**E2**	24	unknown	2012	Medical service II at CHU of city A for 2 years	None
**E3**	25	Single No children	2012	Medical service III at CHU of city A for 3 years	3 days of institutional training in the palliative approach
**E4**	26	Domestic partner No children	2011	One year in private healthcare, Medical service III at CHU of city A for 3 years	none
**E 5**	32	Unknown	2004	Has worked in Medicine, neurology. Surgery I at CHU of city A for 7 years	none
**E6**	26	Single No children	2011	Interim for numerous months Medical service IV at CHU of city A for 3 years	none
**E 7**	25	Domestic partner No children	2015	Medical services I at CH of city B for 9 months	none
**E 8**	26	Civil Union, No children	2013	Two years in geriatric hospice Medical services I at CHU of city C for 7 months	University degree in palliative care training in progress
**E 9**		unknown	1989	medical services II at CH of city B for 6 years	Massage certificate in 2 years University degree in palliative care 2012
**E 10**	31	Domestic partner 1 child	2006	Medicine I at CHU of city C for 10 years	University degree in Algology
**F 1**	54	Married No children	2001	Intensive Care Neurosurgery, Algology. Surgery I at CHU of city A for 6 years	University degree in Algology 2006, 3 days of institutional training in the palliative approach
**F 2**	29	Single 1 child	2010	Surgery I at CHU of city A for 5 years	3 days of institutional training in the palliative approach
**F 3**	34 years old	Common law union 1 child	2011	Medical services V at CHU of city A for 5 years	none
**F 4**	28 years old	Civil Union 1 child	2009	Medicine IV at CHU of city A for 6 years	University degree in palliative care training in progress
**F 5**	41 years old	Married 2 children	1995	Medical intern rheumatology, Medicine II at CHU of city A r for 5 years	3 days of hospital training in pain, caregiving, stress-management
**F 6**	28 years old	Single	2010	Oncology nurse Surgery II at CHU of city A for 3 months	University degree in neuro-oncology Hospital Training in pain management
**F 7**	45 years old	Civil Union, 4 children	2009	Long term geriatrics several years, Mobile Palliative Team at CHU of A for 3 years	University degree in palliative and end-of-life care

All are women except F1. The different medical services were made anonymous: Medical I-V and Surgery I and II for hospital of city A, Medical I and II for the hospital of city B, Medical I for hospital of city C

### Categories

Four categories emerged. They are presented here in general present-tense propositions (phenomenological statements) comprising all meaningful elements (sub-themes or properties).

#### The RN role as a witness to patient experiences, serving as a watchful eye for physicians, which in turn, helps in anticipating and clarifying the steps leading to a patient-centered palliative approach

The presence of nurses, in caregiving moments (emotional or personal), facilitate the process of getting to know the patient: *“the patient will tell us things during the night or in moments of grooming or washing”I3*. Nurses are conscious that the transmission of information facilitates better care for the patient: *“Our role is really to communicate the behavior of the patient in general*” *(I2), “It is the nurse’s job [ …*] *to alert the physician” (I1).*

The follow-up by nurses, over a long period of time, facilitates a patient-centered PA, its anticipation and better follow-up, *“as we get to know patients early [in the care process], support is facilitated from the beginning” (I2,I0,I3); “this presence from the beginning allows for traceability”(F7).* For I0, they consider their mission to be “*helping change the mentality”.*

#### The physician’s position regarding the role of the RNs influences the implementation of a palliative approach and the behavior of professional caregivers

The implementation of a patient-centered PA is a sign of the recognition of a nurse’s work, *“when a palliative approach is put into place, that proves that both what the caregivers have said and what the patient has experienced have been taken into account”*(I2). However, I5 stated, *“the role of the nurse in relation to the patients in palliative care depends on the physician”*. The seven nurses in the focus group talked at length about the importance of the physician’s position within the health care services. The discussion of the group centered around the idea of “medical power” which is rooted in a curative paradigm. For F1, F2, F3, and F7: “*within the group of caregivers there is a hierarchy, there are interactions that involve a power struggle*.” F2 added, “*the way we think is different from physicians*”, highlighting the difference in paradigm.

- A medical viewpoint still rooted in a curative paradigm explains inappropriate behaviors and the delayed implementation of a palliative approach.

The medical decision to move from a curative treatment to palliative care is difficult. “*Physicians have difficulty going into this phase of palliative care*”(I4, I6). F4 confirms that this stance delays entering into a PA, “*if physicians are not at this phase, we cannot move forward … they have a hard time abandoning protocol, and from the time when they’re not in power anymore, they feel perturbed*.”

For I1 and F3 the exclusive use of a curative approach leads to inappropriate behavior on the physician’s behalf such as desertion of the patient once treatment options are exhausted, “*physicians are in a curative mindset right up until the end. So, they have a tendency to neglect the patient when s/he enters into palliative care.”* For F6, F2 and F7, “*oncologists would always like to have just that one last chemotherapy, because when the treatments end, that means that they have failed*.” However, 3 nurses explained that this had changed, “*the physician has understood his/her limitations, I believe that the culture is changing now, we’ve really reached a different level*” (F2, F4, F6).

- The misalignment between the nurse’s professional values and the prescribed work leads to their inappropriate behavior and a loss of meaning of care.

“*where things are not going well, is when physicians don’t understand what nurses are saying to them” (I1); “this clinical approach is one that I’m familiar with, but in the end, is not recognized by others” (I6)*. This leads to different types of attitudes: Frustration and anger, *“we are furious, so many emotions in the end, it’s the meaning of care that is lost” (I6)*. A sense of resignation “*we have our own role, but we are obligated to follow the treatment” (I8).* To the extreme misdeed, to keep a patient centered approach. “*When physicians don’t assume that, nurses misbehave or take their own initiative” (I3, I10).*

#### Palliative approach as a reflective process, which is ethical and anticipated, calls for “rethinking care within a team setting” where time is set aside for this patient-centered approach

For I3 and F7 “*the palliative approach is really reflecting on the patient’s care and not the pure caregiving act*”. For I6 *“[it is a] reflective, ethical, anticipated and multidisciplinary step concerning our care, that should include the experience of the patient as much as possible*” and “i*t is a practice in which we reflect more than we are used to on what we have done*” *(I4,I5, I6, I8,I10).*

The ethical dimension was brought up by a number of participants: “*in the palliative approach we let the patient decide what s/he wants, we give them autonomy*”. Meanwhile, I4 and I5 talk about the “*need for training to really get to know the ethical dimension of care! I think that it is what we are missing.*”

- Space and time set aside for conversation is needed for a palliative approach.

The most useful tool to cope with difficulties and strong emotions is to set aside the space and to share experiences in a team setting where each member can receive psychological support from the others: “*to get together in a group to talk, to be able to make time and that everyone can speak their mind freely!”* I5, F2. I6 added: “*regarding problematic patients, team ethical discussion is essential”*

- A palliative approach should be anticipated.

Nurses deplore the resistance that still occurs, surrounding an anticipated palliative approach: “*It would have been necessary to talk about all of that before, we could have anticipated this! This is what is really missing, the preparation in advance and the communication in the team*!” *I1*. For F2, “*A palliative approach is the only reflective process we’d like to put into place, particularly when planning ahead; this is something we’ll question for the rest of our lives.”*

#### The MPCT as seen as the intermediary that facilitates the physician-nurse “balance” and helps nurses reclaim their professional and ethical values within the environment of shared care

- The MPCT creates a liaison, improving communication between nurses and physicians.

For I3, F2 “*this puts a third party between the physicians and us, it creates a balance*”. I6 “*being at the bedside of a patient with the physicians [is what] improves the communication with [him]”.*

- The MPCT facilitates a reflective process about the care being administered, with a new perspective at a different moment in time.

For I4, I9 and F1, the MPCT gives them the opportunity to take their time: “*A perspective from the outside allows us to come down” […*] *“the rhythm is completely different and that’s what I appreciate*”. I1 says *“what is interesting is that the opinion and the perspective of each person is important!”.*

- The MPCT allows for patient-centered shared care through the exchange of experience, knowledge and skills centered around accompanying the patient at the end of his/her life.

For I4, “*With you, we’re able to restore caregiving*”. I11 “*Shared care is the sharing of knowledge and skills*”.

- The MPCT allows for the reclaiming of professional and ethical values.

For I4, I6, I10, the MPCT allows caregiving to be reestablished. “*With you we are able to reestablish care giving and return to our own approach”*. The MPCT additionally allows for nurses to return to their “*core values*” I8.

**Fig. 1:**
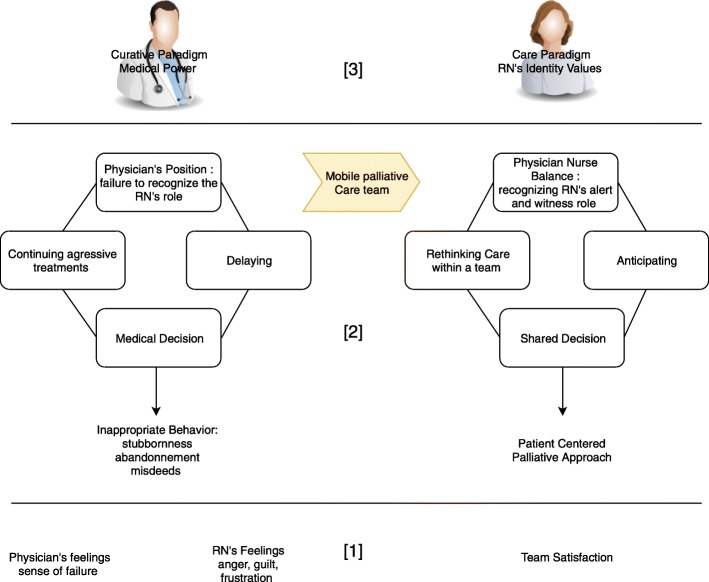
: Semiopragmatic ordering of nurse’s lived experience about palliative approach

Figure 1 Semiopragmatic ordering of RN’s lived experience about palliative approach

## Discussion

Our study clarified our objective to understand the palliative approach of RN in hospital care units and how they make use of the MPCT.

Nurses recognize their role as being witnesses to the patient’s experience through their constant presence which is in line with their professional values. In turn, this gives them an “alert role” that can help in anticipating and preparing for a patient centered palliative approach, in accordance with recent results published by Schroeder & Lorenz [22].

However, in reality, it is the physician’s positioning and his/her attitude toward a palliative approach that sets the tone for its implementation and determines the behavior of different staff members within the healthcare service. According to RNs, when physicians remain anchored in a curative paradigm, there is a risk of not recognizing nurse’s individual role and delaying access to palliative approach. However, Fox et al. [23] pointed to “becoming palliative” is not a defined event; as such, ambiguity and tension contribute to the difficulties involved in negotiating the transition to palliative care. Failure to recognize RN’s role can distort professional values, lead to suffering, inappropriate behavior, or even certain infringement of the rules. Moreno-Milan et al. [24] brought to light the concept of “meaning of work” as a support to personal protective factors (self esteem) and identity values and decreasing perceived stress. This entails striking a balance between the feeling of being inadequate/dissatisfied and adequate/satisfied with one’s work [25]. Studies concerning district nurse’s experiences of palliative care in the home revealed that they experienced a feeling of professional inadequacy [26]. In a recent scoping review, half of the 25 included studies provided possible outcomes of “bad deaths” such as palliative care not being initiated, interpersonal and team conflict [27]. The benefit of this work is to put forward the core category “the recognition of the individual role of the RN” as an essential concept for understanding what causes the delay in the implementation of a PA. This confirms the findings of Reimer-Kirkham et al. [28] and Hahne et al. [29]: the perceived differences in knowledge and skill among team members and a perceived absence of fairness negatively influenced overall cohesion and confidence. Although recognizing a pivotal role of RN in end-of- life care allows to shift from the model of the physician alone deciding to an approach where nurses also shared responsibility [30], several international studies found that the profession is not granted sufficient recognition and legitimacy as shown by Sekse et al. [31]. While physicians planned the care, the nurses, who spent the most time with, and knew the patients the best, were not involved in the planning [32]. Physicians did not listen to the nurses and did not want to prescribe in accordance with their advice [33]. Findings concerning nurses’ role from Hospice in the USA demonstrated poor collaboration with the physicians [34].

Although worldwide training on palliative care for health professionnals is often limited [35], interprofessional training (physicians and nurses) could optimize sharing expertise [36]. This is in line with the 76th World Health Assembly focusing on strengthening palliative care [37] and a recent study showed how nursing homes can become useful sites for learning about end-of-life care during internships [38]. A collective training program (undergraduate and postgraduate) for medical and nurse students would give an opportunity to learn from and about each other, which facilitate this recognition of each person’s skills as well as a clarification of the individual role.

The hypothesis that the questioning of RN professional values limiting his/her capacity to become involved in palliative care could explain the results of the work that highlight the reluctance despite training [15, 16] or those showing difficulties in partnering with a MPCT [10]. In contrast to what the palliative care nurses reported about their interventions at CHU of Montpellier, those in our study indicated a need for the use of MPCTs and their important contribution on a personal and professional level. They considered them as a “facilitating intermediary” within the healthcare service restoring a “balance” in the communication and in decision-making between physicians and other care workers, leading care teams to an anticipated and patient-centered palliative approach with regards to roles and skills according to guidelines. It is possible that for the RNs in our sample, the interventions from the MPCTs helped them recover their professional values as nurses and diminish the reluctance.

### Strengths and weaknesses of the study

This work hypothesized an internal methodological consistency between the object of research (lived experience), the data collection methods, and the data analysis favouring a logic of emergence. In each step of the process, there was transparency.

Regarding the data collection, the nurses of the MPCT that conducted the interviews were known by most of the participants in the study. This may introduces a bias. However, at the same time, this also allows for an environment of confidence and authenticity in the responses. It was difficult to find times when nurses were available because the interviews happened during work hours and it was necessary to get approval from the administrators and physicians in their area.

Most of the nurses interviewed were young (*n* = 15 less than 40 yo) with little to no training in palliative care (*n* = 14 less than 3 days training). Regarding the situation in France, hospital nurses are younger than non-hospital and freelance nurses. This may be a consequence of legislation requiring newly graduated nurses to practice under the authority of a head nurse. One hypothesis could be that an activity primarily focused on curative care in these care units does not make palliative care training a priority. The interviews were stopped upon reaching data saturation. The triangulation of methods of data collection (individual interviews and focus group), as well as the triangulation of analysts (5 researchers discussing their results) permitted for more nuanced information and brought validity to the results. The individual interviews gave personal and private information about the feelings of the nurses. The focus group generated information focused on their professional position, medical power and the common feeling of lack of recognition. Additionally, the focus group carry out the opposing positions of the two paradigms: that of the nurses, which is more focused on care and that of the physicians, which is more directed toward the cure. A similar study on physicians is in its final stages, would nuance this results.

The originality of this semiopragmatical analysis is that it is the only one allowing a logical ordering of categories.

## Conclusion

We have shown that the physician-nurse balance, recognizing the individual role of the nurse facilitates the initiation of a patient-centered PA which is anticipated, while the opposite can lead to a delay as well as inappropriate behavior. A time set aside for interdisciplinary exchange leads parties involved to “rethink care” with regards to roles and skills of physicians and nurses. Nursing experts on the MPCT contribute to this reflective process by bringing personal and professional assistance to nurses within the healthcare system and finding again ethical values.

## Data Availability

The datasets used and analyzed during the current study are available from the corresponding author on reasonable request.

## Abbreviations

CHU: Centre Hospitalier Universitaire
MPCT: Mobile Palliative Care Team
PA: Palliative Approach
PC: Palliative Care
RN: Registered Nurses
